# Degradation characteristics, cell viability and host tissue responses of PDLLA-based scaffold with PRGD and β-TCP nanoparticles incorporation

**DOI:** 10.1093/rb/rbw017

**Published:** 2016-04-08

**Authors:** Jiling Yi, Feng Xiong, Binbin Li, Heping Chen, Yixia Yin, Honglian Dai, Shipu Li

**Affiliations:** ^1^State Key Laboratory of Advanced Technology for Materials Synthesis and Processing, Wuhan University of Technology, Wuhan 430070, China;; ^2^School of Medicine, University of Arizona, Tucson, AZ 85721, USA

**Keywords:** PDLLA/PRGD/β-TCP scaffold, cell viability, degradation, host tissue responses

## Abstract

This study is aimed to evaluate the degradation characteristics, cell viability and host tissue responses of PDLLA/PRGD/β-TCP (PRT) composite nerve scaffold, which was fabricated by poly(d, l-lactic acid) (PDLLA), RGD peptide(Gly-Arg-Gly-Asp-Tyr, GRGDY, abbreviated as RGD) modified poly-{(lactic acid)-co-[(glycolic acid)-alt-(l-lysine)]}(PRGD) and β-tricalcium phosphate (β-TCP). The scaffolds’ *in vitro* degradation behaviors were investigated in detail by analysing changes in weight loss, pH and morphology. Then, the 3-(4,5-dimethyl-2-thiazolyl) -2,5-diphenyl-2 -H-tetrazolium bromide (MTT) assay and cell live/dead assay were carried out to assess their cell viability. Moreover, *in vivo* degradation patterns and host inflammation responses were monitored by subcutaneous implantation of PRT scaffold in rats. Our data showed that, among the tested scaffolds, the PRT scaffold had the best buffering capacity (pH = 6.1–6.3) and fastest degradation rate (12.4%, 8 weeks) during *in vitro* study, which was contributed by the incorporation of β-TCP nanoparticles. After *in vitro* and *in vivo* degradation, the high porosity structure of PRT could be observed using scanning electron microscopy. Meanwhile, the PRT scaffold could significantly promote cell survival. In the PRT scaffold implantation region, less inflammatory cells (especially for neutrophil and lymphocyte) could be detected. These results indicated that the PRT composite scaffold had a good biodegradable property; it could improve cells survival and reduced the adverse host tissue inflammation responses.

## Introduction

Peripheral nerve injury, which is frequently encountered in daily life, would cause serious health problems to patients. Although injured peripheral nerve could regenerate spontaneously, the regenerative capacity is limited by long-distance defect [1, 2]. Currently, the most popular medical technique for nerve repair is using autologous nerve to bridge the gap [3, 4]. While the source of autologous tissue is very limited, a second surgery was always needed and some donor site morbidities might be induced during the autologous graft [5, 6]. As a promising alternative to provide the mechanical support, more and more natural or synthetic biodegradable artificial scaffolds were used for the recovery of severed nerve, such as collagen, chitosan, silk, polyglycolic acid and Poly (d, l-lactic acid) (PDLLA) [7–9]. Thereinto, PDLLA had been approved for clinical use by FDA (Food and drug administration), due to its good solubility, mechanical and biodegradable properties [10]. However, the PDLLA had a slow biodegradation rate and a poor cyto-compatibility, because its degradation products were acid, by which the aseptic inflammation and heterologous responses were often triggered [11, 12], the common adverse effects were depression of nerve regeneration, tissue necrosis and the loss of sensory and motor function [13]. According to the principles of evaluations of scaffolds to be used for peripheral nerve repair, the ideal scaffold is not only able to guide the elongation of nerve axon, but also degradable, biocompatible and reduces inflammation responses [14]. So PDLLA scaffolds need to be further explored and modified.

To achieve a better performance, our group chose PDLLA as the substrate material and incorporated with RGD peptide (Gly-Arg-Gly-Asp-Tyr, GRGDY, abbreviated as RGD) and β-tricalcium phosphate (β-TCP) nanoparticles to synthesis a composite nerve scaffold Poly (**d**, **l**-lactic acid)/RGD peptide modification of poly{(lactic acid)-co-[(glycolic acid) -alt-(**l**-lysine)]}/β-tricalcium phosphate (PDLLA/PRGD/β-TCP, abbreviated as PRT). Each of these components could provide positive influences on the scaffold. It was proved that RGD peptides could enhance the nerve cell attachment, elongation and facilitate axon growth *in vivo* [15, 16]. Moon *et al.* [17] also found that the RGD peptide was able to attenuate inflammatory responses by inhibiting integrin signaled mitogen-activated protein kinase pathways, which play an important role in nerve recovery. Additionally, the β-TCP was usually used in bone repair and exhibited a good biocompatibility. It was also desired to neutralize the acid of degradation products of PDLLA [18, 19]. Besides that, calcium ion was reported as a crucial factor affecting axonal outgrowth and the elongation of nerve growth cone [20]. Previous studies have revealed that PRT scaffolds had a good biocompatibility [21–24].

However, deeply researches were needed to judge whether PRT scaffold were suitable for clinical practice, especially, the degradation characteristics, cell viability and host tissue responses of the PRT scaffold should be systematically explored. In this study, we synthesized the PRT composite scaffold along with three other scaffolds PDLLA (P), PDLLA/PRGD (PR) and PDLLA/β-TCP (PT). Following the comparative studies reported herein, (i) the degradation characteristics consisting of weight loss, pH change and morphology, (ii) cell viability concerning of cell survival and cell proliferation and (iii) host inflammatory responses were, respectively, evaluated.

## Materials and Methods

### Materials

PDLLA (Molecular weight = 250 000) and β-TCP nanoparticles were synthesized in our laboratory. The following chemicals were derived from shown sources: RGD Peptide (GL Biochem); 1, l′-carbonyldiimidazole (CDI, GL Biochem); 1-ethyl-3-(3-dimethylaminopropyl) carbodiimide (EDAC, Aldrich); Apoptosis, Necrosis Assay Kit (Beyotime, China). Other chemicals were analytical or reagent grade. PC12 cells were derived from Shanghai Institute of Cell Biology. SD rats were obtained from Hubei provincial center for disease control and prevention.

### Scaffold preparation

The following steps were used to synthesize the composite PRT scaffold with RGD andβ-TCP nanoparticles incorporation. First, (3S)-3-[4-(benzyloxycar bonylamino) butyl] morpholine-2, 5-dione (BMD) was synthesized by bromoacetyl bromide and Ne-(benzyloxycarbonyl)-l-lysine. Second, poly {(lactic acid)-co-[(glycolic acid)-alt- (Ne-benzyloxycarbonyl-l-lysine)]} (PLGLZ) was obtained by copolymerization of d, l-lactide and BMD. Then, poly {(lactic acid)-co-[(glycolic acid)-alt-(l-lysine)]} (PLGL) was synthesized by catalytic hydrogenation of PLGL. Finally, PLGL was modified with RGD peptide. PRGD (0.05 g) and PDLLA (0.9 g) was dissolved in ethyl acetate at a concentration of 5 wt %.β-TCP (0.05 g) was added to ethyl acetate solution and mixed thoroughly. PDLLA/PRGD/β-TCP composite was prepared by using solvent evaporation method. The PDLLA/PRGD/β-TCP, PDLLA/β-TCP, PDLLA/PRGD and PDLLA were fabricated to scaffolds. The nerve scaffolds were sterilized with ultraviolet light for subsequent experiments. The methods for preparation and characterizations of these scaffolds have been previously described [25].

### *In vitro* degradation assay: pH change and weight loss

Four different types of scaffolds (P, PR, RT and PRT) were prepared and tested. These scaffolds were placed in normal saline solution (1 mg/ml, scaffold to solution ratio) and incubated for 8 weeks at 37°C under shaking condition (100 rpm). With the incubation of scaffolds in saline solution for 2 h, an initial pH was measured. Thereafter, the pH of the saline solution was measured with pH meter (PHSJ-3F, Shanghai INESA&Scientific Instrument Co. Ltd), at the end of each week during the 8-week incubation. Upon pH test, the scaffolds were washed with distilled water (three times) and vacuum-dried for 1 week (room temperature), before weight measurement by Precision electronic balance (AUW120, Shimadzu Corporation, Japan). The mass change was determined by the equation: wt% = 100× (Wr − W_0_)/W_0_, where W_0_ was original weight, and Wr was dried weight [26].

### Morphology analysis

Following the incubation for 8 weeks and dried in vacuum, the morphological changes of these scaffolds (P, PR, RT and PRT) were recorded for by scanning electron microscopy (SEM) (JSM-5900LV, Japan).

### Cell viability of PRT scaffold

#### Cell proliferation

PC12 cells were cultured and maintained in F12K medium containing 10% fetal bovine serum and 5% equinum serum (Gibico, USA). For the assay, the PC12 cells (4000/well) were cultured in 96-well plates with 200 μl medium per well. Then, 20 μl scaffold-incubated saline solution [the samples (P, PR, PT, PRT) were collected at the first week] was added to cell culture. Following cell growth for Days 1, 3, 5 and 7, 3-(4,5-dimethyl-2-thiazolyl) -2,5-diphenyl-2 -H- tetrazolium bromide (MTT, 5 mg/ml), was added to cell culture(20 μl/well) with incubation for 4 h at 37°C. Then the medium was removed and dimethylsulfoxide was added (150 μl/well) with sufficient mixing. The absorbance at 450 nm was measured with an enzyme-linked immunosorbent assay reader (Thermo Fisher Scientific, Finland).

#### Cell live/dead assay

PC12 cells (2000/well) were cultured in 96-well plates for 7 days, with medium containing 20 μl scaffold-incubated saline solution [the samples (P, PR, PT, PRT) were collected at the first week, as above]. To determine live-dead cell population, the cells were stained with 1 mM propidium iodide (dead cells were labeled red) and 5 mM Hoechst 33342 (live cells were labeled blue) for 30 min at room temperature [27, 28], then observed by a fluorescence microscope (Olympus, IX71, Japan).

#### *In vivo* degradation and histological assessment

All procedures undertaken in this study were approved by the Animal Care and Use Committees of Wuhan University of Technology and conformed to National Institutes of Health guidelines. Based on the *in vitro* degradation data, we chose P and PRT scaffolds for histology evaluation. Twelve adult male SD rats (180–200 g) were averagely and randomly divided into two groups (P, PRT) and anesthetized with 50 mg/kg body weight pentobarbital sodium. After their backs were shaved and sterilized with Betadine, two pieces of scaffolds were implanted in subcutaneously in each rat as previously described [29]. At 8 weeks post-implantation, the scaffolds were retrieved for SEM analysis. The subcutaneous tissue of each group were harvested and fixed with paraformaldehyde (4%) overnight. Then, the specimens were dehydrated with increasing gradient concentration of ethanol, embedded in paraffin and cut into 4 µm thickness sections. A hematoxylin and eosin (H&E) stain was applied before the tissue morphology was evaluated by an inverted microscopy (Olympus, IX71, Japan) and average number of inflammatory cells in each group was counted [30].

#### Statistics analysis of data

Statistical analysis of data was performed with one-way analysis of variance followed by a *t*-test; Statistical significance was defined as *P* values <0.05. Data are presented as mean ± standard error.

## Result and Discussion

### Degradation characteristics of PRT scaffold *in vitro*

**Figure 1.**
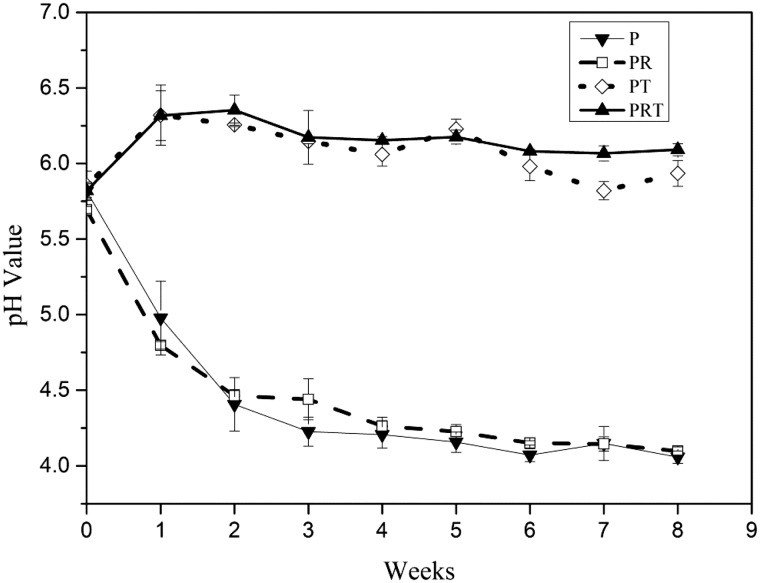
PH changes of P, PR, PT and PRT scaffolds degraded *in vitro*. (P: poly(d,l-lactic acid); PR: poly(d,l-lactic acid)/RGD peptide modification of poly{(lactic acid)-co-[(glycolic acid) -alt-(l-lysine)]}; PT: poly(d,l-lactic acid)/β-tricalcium phosphate; PRT: poly(d,l-lactic acid)/RGD peptide modification of poly{(lactic acid)-co-[(glycolic acid) -alt-(l-lysine)]}/β-tricalcium phosphate).

To evaluate the degradation of PRT scaffold, an 8 week incubation in normal saline were carried out, and the pH value were measured weekly with an initial test at 2 h. After first 2 h degradation, the pH values of all tested scaffold dropped from 6.2 to 5.5 approximately (Fig.1). Then pH results at each week showed varied trends. For scaffolds P and PR, the pH of saline solution dropped quickly (<4.5) within initial 2 weeks and slowly reach 4.0 at the end. On the contrary, the pH of PT and PRT scaffolds rose quickly (5.8–6.3) in the first week and maintained similar level (6.0–6.3) in the subsequent weeks (2–8). These data indicated the β-TCP nanoparticles, as an incorporated component, could neutralize the acid products released from PDLLA degradation. This was expected to notably reduce the unwanted effect of the scaffold when used in animal implantation tests, even human tissue regeneration in the future.

**Figure 2.**
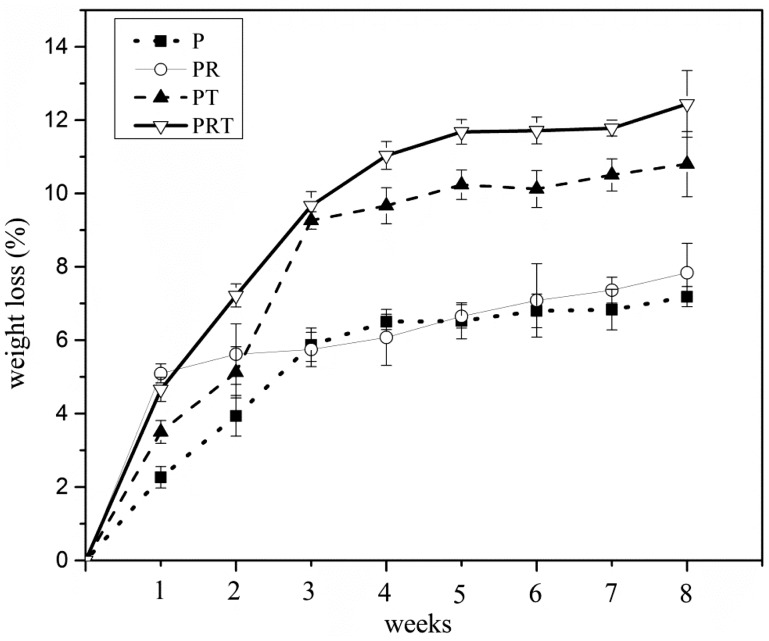
Weight loss ratio of P, PR, PT and PRT scaffolds degraded *in vitro*. (P: poly(d,l-lactic acid); PR: poly(d,l-lactic acid)/RGD peptide modification of poly{(lactic acid)-co-[(glycolic acid) -alt-(L-lysine)]}; PT: poly(d,l-lactic acid)/β-tricalcium phosphate; PRT: poly(d,l-lactic acid)/RGD peptide modification of poly{(lactic acid)-co-[(glycolic acid) -alt-(l-lysine)]}/β-tricalcium phosphate).

**Figure 3.**
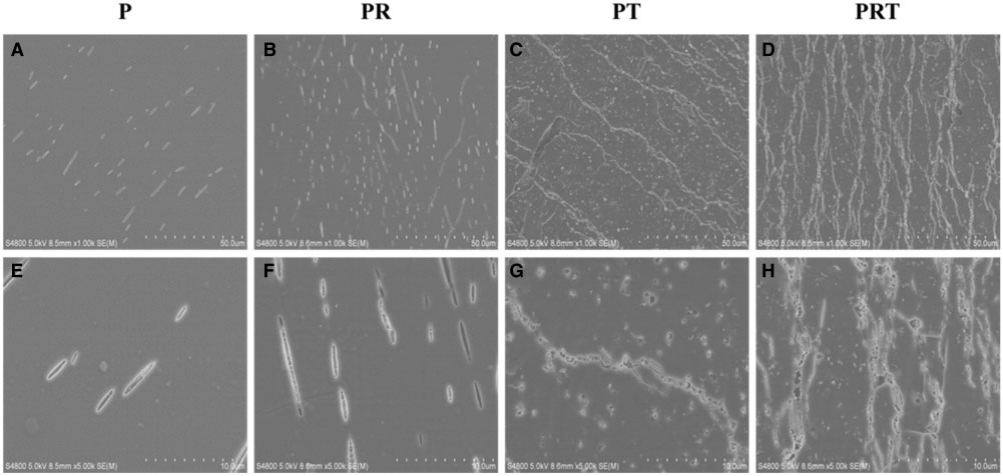
Morphology of P, PR, PT and PRT scaffolds degraded *in vitro*. (Scale bars: 50 μm in A–D, 10 μm in E–H. **P**: poly(d,l-lactic acid); **PR**: poly(d,l-lactic acid)/RGD peptide modification of poly{(lactic acid)-co-[(glycolic acid) -alt-(l-lysine)]}; **PT**: poly(d,l-lactic acid)/β-tricalcium phosphate; **PRT**: poly(d,l-lactic acid)/RGD peptide modification of poly{(lactic acid)-co-[(glycolic acid) -alt-(l-lysine)]}/β-tricalcium phosphate).

Meanwhile, the weight losses of the scaffolds were assessed. As illustrated in Fig. 2, the weight loss in the initial week was 2.2%, 5.1%, 3.5% and 4.7%, respectively, for scaffolds P, PR, PT and PRT. In the subsequent weeks, varied trend had also been demonstrated. At the end of 8 weeks, maximum weight loss was reached (7.2%, 7.8%, 10.8% and 12.4%, respectively, for the scaffolds P, PR, PT and PRT). Compared with other scaffolds, PRT scaffold had the best degradation rate. We speculated that its thickness and β-TCP nanoparticles were the two most important factors, according to Grizzi’s theory, the ideal thickness for PDLLA degradation should be 200–300 μm and this parameter of PRT was in the range (200 μm) [31]. For the β-TCP nanoparticles, the hydrophilicity of PDLLA was enhanced by its incorporation, and its dissolution would accelerate the degradation of PRT scaffold in the slightly acidic environment, which was created by the degradation of PDLLA. Until the 8 weeks incubation was done, SEM was employed to observe the changes in morphologies of the scaffolds. As shown in Fig. 3, there were distinct morphological forms among the four scaffolds examined, consistent with the weight loss feature present above. Scaffolds PRT and PT displayed a morphological form with higher porosity than those of PR and P, which was mainly contributed by the incorporations of RGD peptides and β-TCP nanoparticles.

### Cell viability of PRT scaffold

**Figure 4.**
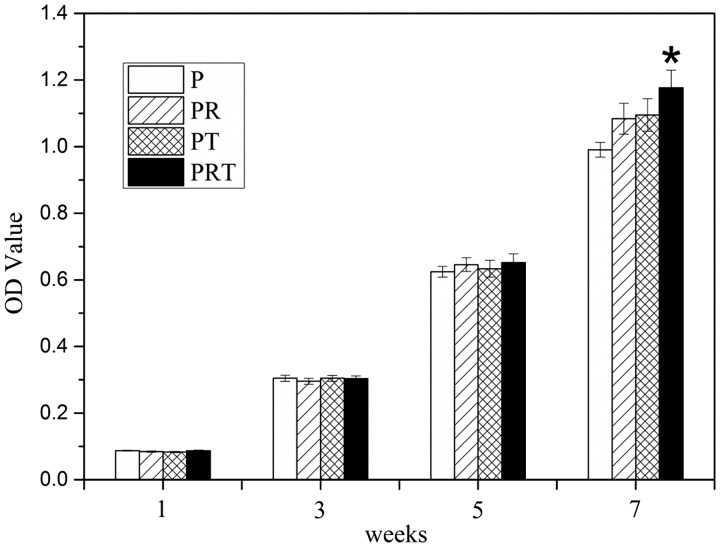
PC12 cell viability cultured in the degradation liquid of P, PR, PT and PRT scaffolds. (**P**: poly(d,l-lactic acid); **PR**: poly(d,l-lactic acid)/RGD peptide modification of poly{(lactic acid)-co-[(glycolic acid) -alt-(l-lysine)]}; **PT**: poly(d,l-lactic acid)/β-tricalcium phosphate; **PRT**: poly(d,l-lactic acid)/RGD peptide modification of poly{(lactic acid)-co-[(glycolic acid) -alt-(l-lysine)]}/β-tricalcium phosphate)

**Figure 5.**
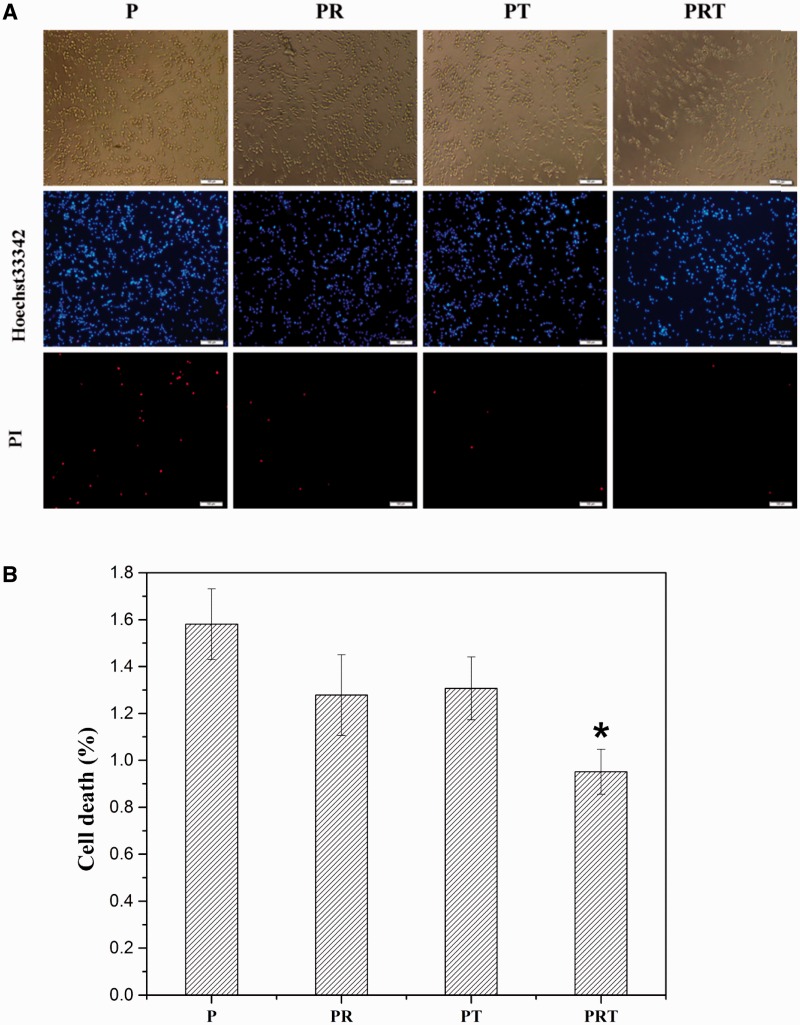
PI/Hochst33342 staining (A) and cell death ratio (B) after cultured in the degradable solution of P, PR, PT and PRT scaffolds. (*P < 0.05, compared with P scaffold. **P**: poly(d,l-lactic acid); **PR**: poly(d,l-lactic acid)/RGD peptide modification of poly{(lactic acid)-co-[(glycolic acid) -alt-(l-lysine)]}; **PT**: poly(d,l-lactic acid)/β-tricalcium phosphate; **PRT**: poly(d,l-lactic acid)/RGD peptide modification of poly{(lactic acid)-co-[(glycolic acid) -alt-(l-lysine)]}/β-tricalcium phosphate)

Then MTT assays and staining assays were undertaken to investigate the scaffolds’ (P, PR, PT and PRT) effects on cell viability in terms of cell proliferation and live/dead counts. The pheochromocytoma derived cells (PC12) was chosen and cultured in medium containing 10% scaffold-incubated saline for 7 days. As shown in Fig. 4, the growth of PC 12 cells seemed to maintain a similar lever at Days 1, 3 and 5 for all tested group; however, the cell proliferation cultured in PRT-scaffold incubated saline was notably higher than those of other scaffold-incubated saline at Day 7, particularly that of P-scaffold incubated saline (*P < 0.05*). Hochst33342 and propidium iodide staining results were shown in Fig. 5A and B. Majority of the cells were alive (blue), whereas the lowest ratio of dead cells (red) could be observed in the PRT scaffold group (*P < *0.05). These results suggested that PRT scaffold could promote PC12 cell survival and prevent cell death.

### Morphology of PRT scaffold implanted *in vivo* and inflammatory responses

**Figure 6.**
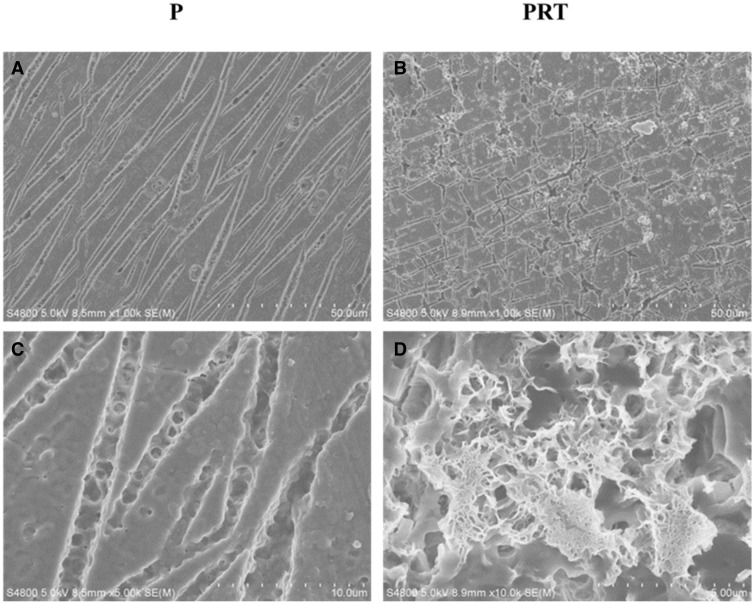
Morphology of P and PRT scaffolds degraded *in vivo*. (Scale bars: 50 μm in A and B, 10 μm in C and D. **P**: poly(d,l-lactic acid); **PRT**: poly(d,l-lactic acid)/RGD peptide modification of poly{(lactic acid)-co-[(glycolic acid) -alt-(l-lysine)]}/β-tricalcium phosphate)

Based on the results of *in vitro* studies, PRT and P scaffold were chosen in *in* *vivo* researches. Their morphology, degradation, as well as host tissue regeneration and inflammation responses were compared. After being implanted, a tubular pattern with larger empty areas could be demonstrated in the P scaffold (Fig. 6A and C), while the degradation of PRT scaffold seemed to be uniform. In addition, small pores could be observed on the surface of PRT scaffold (Fig. 6B and D). It was worth noting that the size of pore was larger than that generated by *in* *vitro* degradation, which might be influenced by multiple factors, such as tissues and body fluid.

**Figure 7.**
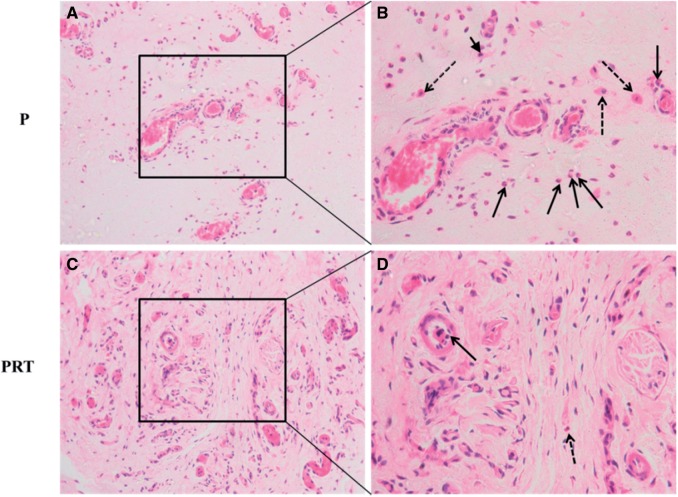
HE staining of subcutaneous tissue after P and PRT scaffolds implantation for 8 weeks. (Solid arrow: neutrophils; arrow head: lymphocytes; dotted arrow: monocytes. (A) and (C) Scale bar =50 μm; (B) and (D) Scale bar =20 μm. **P**: poly(d,l-lactic acid); **PRT**: poly(d,l-lactic acid)/RGD peptide modification of poly{(lactic acid)-co-[(glycolic acid) -alt-(l-lysine)]}/β-tricalcium phosphate).

**Figure 8.**
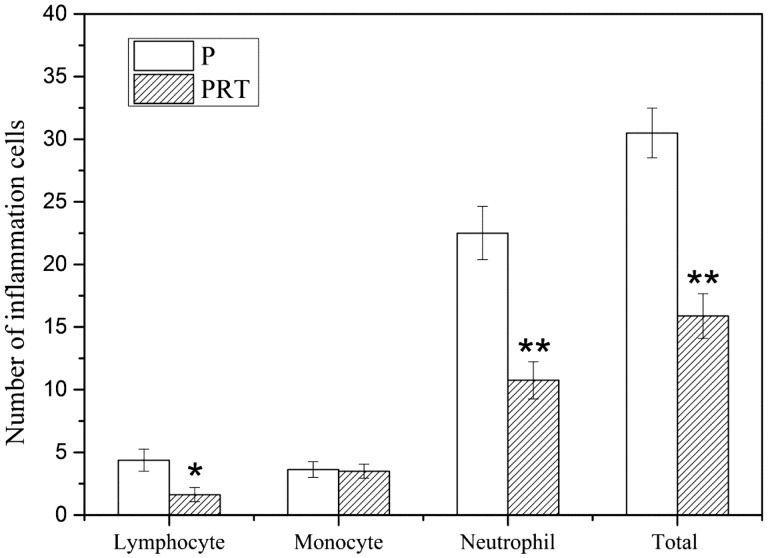
The number of inflammation cells after P and PRT scaffolds implantation for 8 weeks. (*P < 0.05, **P < 0.01. **P**: poly(d,l-lactic acid); **PRT**: poly(d,l-lactic acid)/RGD peptide modification of poly{(lactic acid)-co-[(glycolic acid) -alt-(l-lysine)]}/β-tricalcium phosphate).

Concerning the host tissue responses, the wound healing in all implanted rats have preceded relatively well without any apparent infection and there were more dense tissues in the implanted site of PRT scaffold than P scaffold. As a foreign agent, implantations of scaffolds in subcutaneous tissue would cause inflammation reaction by various degrees, which inevitably affect scaffolds’ biocompatibility [30, 32]. To examine the level of inflammatory responding triggered by PRT and P scaffolds, subcutaneous tissues were sectioned and stained by H&E. It is evident that some inflammatory cells consisting of neutrophils, monocyte and lymphocytes were exhibited in P group (Fig. 7B) and PRT group (Fig. 7D). Subsequently, these inflammatory cells were, respectively, counted from 10 randomly selected areas (Fig. 8) and numbers were analysed, it is found that the numbers of monocytes of two groups were nearly the same, but numbers of lymphocyte (*P <* 0.05) and neutrophils (*P <* 0.01) in PRT group were much lower than those in P group. All these data indicated that the incorporation of β-TCP and RGD could reduce host inflammatory responses leaded by PDLLA scaffold. As an alkaline substance, β-TCP could neutralize degradation products which will easily trigger aseptic inflammation, and RGD, as a good non-immunogenic peptide, could interact with cell membrane proteins to promote cell adhesion and growth or competitively bind integrin receptors of cytomembrane to ameliorate inflammatory cascades [33–35].

## Conclusion

In this study, we have synthesized a composite scaffold PDLLA/PRGD/β-TCP(PRT) and investigated the PRT scaffold properties concerning the degradation, cell viability and host tissue responses, in comparison to much simpler form of the scaffolds (P, PR and PT). Collectively, our data show that the composite PRT scaffold displays several desired properties due to incorporation of RGD and β-TCP: (i) ability to maintain the relative stable pH during its degradation; (ii) relatively fast degradation with uniform morphological change and (iii) capacity to enhance cell (PC12) survival *in vitro* and increased tissue regeneration and reduced inflammation responses *in vivo*. In summary, the composite PRT scaffold has several excellent features including biocompatibility and histocompatibility. This composite PRT scaffold may be widely applied for tissue regeneration upon further and expanded studies.

## Supplementary Material

Supplementary data
