# Supplementary material for: Degradation characteristics, cell viability and host tissue responses of PDLLA-based scaffold with PRGD and β-TCP nanoparticles incorporation
Source: Regen Biomater. 2016 Apr 8;3(3):159–66. doi: 10.1093/rb/rbw017 (PMC4881616; doi:10.1093/rb/rbw017)
Supplement: Supplementary data [file rb_rbw017_index.html]

Supplementary Data | Regenerative Biomaterials

## Supplementary Data

files

- Supplementary Data - pdf file
