# Supplementary material for: Degradation characteristics, cell viability and host tissue responses of PDLLA-based scaffold with PRGD and β-TCP nanoparticles incorporation
Source: Regen Biomater. 2016 Apr 8;3(3):159–66. doi: 10.1093/rb/rbw017 (PMC4881616; doi:10.1093/rb/rbw017)
Supplement: Supplementary data [file 8524c0a572d2e3cf0532a8ea416c4722_rb-2016-008.pdf]

Supplements

Table.S1 Full names of tested scaffolds

Abbreviation	Full name
PDLLA(P)	poly(D,L-lactic acid)
PDLLA/β-TCP(PT)	poly(D,L-lactic acid)/β-tricalcium phosphate
PDLLA/PRGD(PR)	poly(D,L-lactic acid)/ RGD peptide modification of poly{(lactic acid)-co-[(glycolic acid)-alt-(L-lysine)]}
PDLLA/PRGD/β-TCP(PRT)	poly(D,L-lactic acid)/ RGD peptide modification of poly{(lactic acid)-co-[(glycolic acid)-alt-(L-lysine)]}/β-tricalcium phosphate

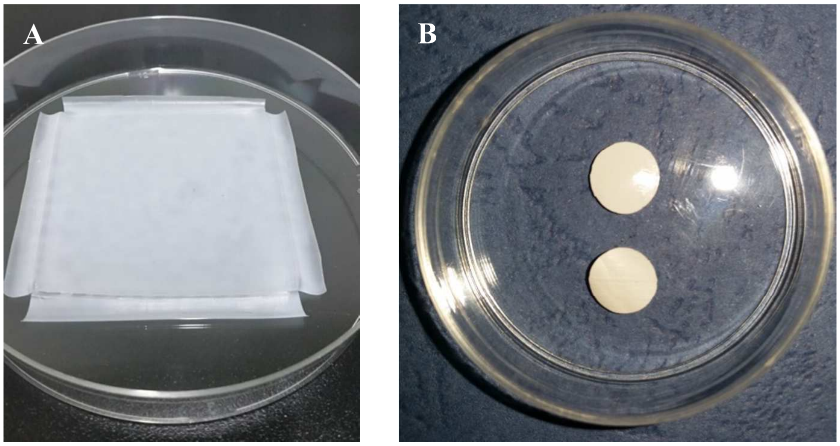

Fig S1.The forms of PRT scaffold.
